# Experiences of land-based treatment program for Indigenous people: a mixed-methods study of Gwekwaadziwin Miikan

**DOI:** 10.17269/s41997-025-01131-4

**Published:** 2026-02-09

**Authors:** Nav Persaud, Hannah Woods, Aine Workentin, Areesha Sabir, Julie Wilder, Godfrey Shawanda, Andrew Aguonie, Matt Maracle, Sam Gilchrist, Anne-Marie Thibault

**Affiliations:** 1 MAP Centre for Urban Health Solutions, Li Ka Shing Knowledge Institute, St. Michael’s Hospital, Unity Health Toronto, Toronto, ON Canada; 2 Department of Family and Community Medicine, University of Toronto, Toronto, ON Canada; 3 Department of Family and Community Medicine, St Michael’s Hospital, Toronto, ON Canada; 4 Gwekwaadziwin Miikan, Little Current, Canada

**Keywords:** Indigenous peoples, Mental health services, Holistic health, Program evaluation, Peuples autochtones, Services de santé mentale, Santé holistique, Évaluation de programme

## Abstract

**Objectives:**

Land-based treatment programs for mental health and addictions challenges may counteract the negative effects of colonization on Indigenous peoples by centring First Nations cultures and approaches to healing that are tied to the land. The purpose of this mixed-methods study was to describe the experiences of participants in the Gwekwaadziwin Miikan land-based mental health and addictions treatment program.

**Methods:**

This mixed-methods study was co-designed with treatment program participants and based on input from the United Chiefs of Mnidoo Mnising and the Elder Council and Knowledge Keepers. Semi-structured in-person one-on-one interviews were conducted during the 90-day land-based treatment program and 6 months after the end of the land-based treatment.

**Results:**

We completed 39 in-person interviews during treatment and 20 6-month follow-up interviews by telephone in addition to attending circles and other group activities. Four overarching themes were identified: connection with Indigeneity, connection with the land, trauma, and peer relationships. All 20 participants completing the follow-up interview reported not using substances at 6 months. Five (25%) reported relapses at some point after leaving the program but had since stopped using.

**Conclusion:**

The Gwekwaadziwin Miikan land-based treatment program provided connection with Indigeneity and with the land that might counteract processes that dispossess Indigenous people of their land. These findings can help support the wider implementation of land-based treatment programs and similar programs that centre Indigenous learnings and healing practices.

**Supplementary Information:**

The online version contains supplementary material available at https://doi.org/10.17269/s41997-025-01131-4.

## Résumé

**Objectifs:**

Les programmes de traitement sur le territoire des problèmes de santé mentale et des dépendances font contrepoids aux effets néfastes de la colonisation sur les personnes autochtones en se concentrant sur les cultures des Premières Nations et sur les stratégies de guérison liées au territoire. Nous avons cherché à décrire les expériences des personnes ayant participé à Gwekwaadziwin Miikan, un programme de traitement sur le territoire des problèmes de santé mentale et des dépendances.

**Méthodes:**

Conçue en collaboration avec les personnes ayant participé au programme, notre étude à méthodes mixtes était aussi fondée sur les apports des Chefs unis, du Conseil des aînés et des gardiens et gardiennes du savoir de Mnidoo Mnising. Des entretiens semi-directifs en personne individualisés ont été menés au cours des 90 jours du programme de traitement sur le territoire et 6 mois après la fin du traitement sur le territoire.

**Résultats:**

Nous avons mené 39 entretiens directs au cours du traitement et 20 entretiens de suivi par téléphone 6 mois après, en plus d’assister à des cercles et à d’autres activités de groupe. Nous en avons dégagé quatre grands thèmes: le rapport à l’indigénéité, le rapport au territoire, les traumatismes et les relations avec les pairs. Les 20 personnes participantes s’étant prêtées aux entretiens de suivi ont déclaré ne pas avoir consommé de substances après 6 mois. Cinq (25%) ont déclaré avoir fait des rechutes après la fin du programme, mais avoir cessé de consommer depuis.

**Conclusion:**

Le programme de traitement sur le territoire Gwekwaadziwin Miikan a fourni un rapport à l’indigénéité et au territoire pouvant faire contrepoids au processus de dépossession des personnes autochtones de leurs terres. Les constats de l’étude jouent en faveur de la mise en oeuvre élargie des programmes de traitement sur le territoire et des programmes semblablement axés sur les pratiques d’apprentissage et de guérison autochtones.

**Figure:**
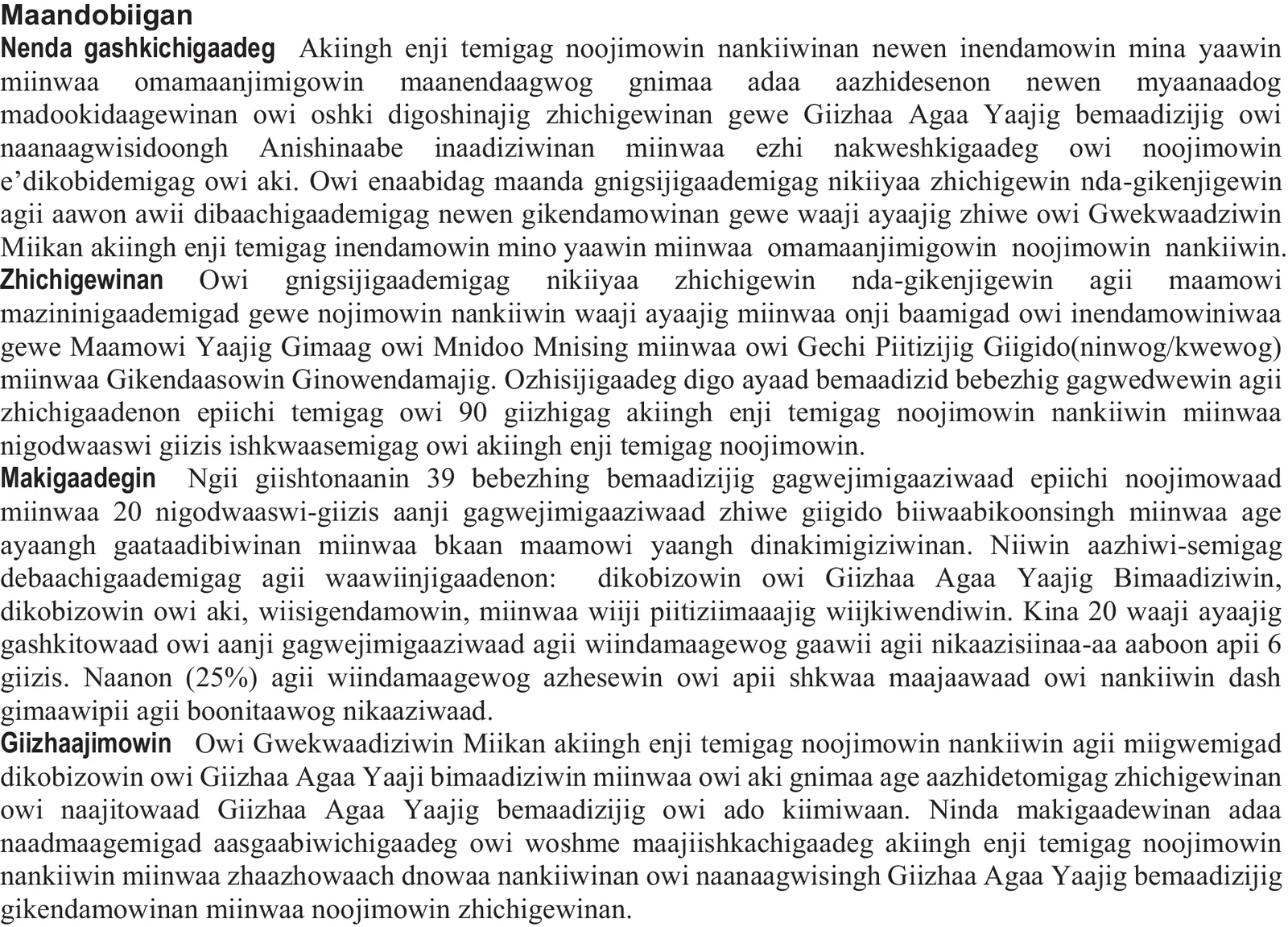

## Introduction

Finding strength through connections with the land is a potential response to the process of colonization that violently separates Indigenous people from their land (Ahmed et al., 2021, 2023a, 2023b; National Inquiry into Missing and Murdered Indigenous Women and Girls (Canada) et al., 2019). As reflected in one participant’s words, “…Reconnecting, rebuilding…just being here, even the conversations or ceremonies, it’ll pop up, stuff that I do already know. It’ll help me remember it”. Land-based treatment programs can reconnect Indigenous people with the land as part of a healing process (Mattick et al., 2014) and address intergenerational trauma, or trauma experienced by Indigenous people harmed by violent colonial processes such as residential schools (Gittings et al., 2024). Land-based treatment programs have been defined as “…a health or healing program or service that takes place in a non-urban, rural or remote location on a land base that has been intentionally spiritually cultivated to ensure the land is honoured and respected. The land is understood to be an active host and partner to the people engaged in the healing process…” (Redvers, 2016). A systematic review of nine studies of land-based treatment programs involving physical activity improved physical health, spiritual health, mental health, and community connections based on qualitative analyses of interviews and focus groups (Ahmed et al., 2021). Connections with nature can improve mental health (Nejade et al., 2022) and are of special importance for Indigenous people (Ahmed et al., 2021; Cunsolo Willox et al., 2012).

Access to treatments for opioid dependence is limited while overprescribing of opioid medicines and the spread of misinformation about them have contributed to a massive increase in the rate of opioid-related harms since the 1990s (Van Zee, 2009). Health care settings can be hostile and dangerous for Indigenous people, especially when seeking care for mental health and substance use challenges (Ahmed et al., 2021, 2023a; Morton Ninomiya et al., 2022; Nelson & Wilson, 2017). Land-based treatment could complement other management options for mental health conditions and substance use that have limited effectiveness and availability (Klimas et al., 2018). Medical treatments for alcohol and opioid use disorders improve some outcomes such as treatment retention and the number of days of heavy substance use, but they do not necessarily reduce substance use in the long term (Jonas et al., 2014; Mattick et al., 2014). In addition to offering medical treatments for Indigenous people who have been harmed by the opioid crisis, it could also make sense to provide access to land-based treatment programs. Clinicians caring for Indigenous people who may benefit from being offered land-based treatment programs may be less aware of land-based treatment programs compared with other treatment modalities.

The Gwekwaadziwin Miikan Mental Health and Addiction Program is a partnership between six First Nations, the Manitoulin Island community, and the Government of Ontario. Gwekwaadziwin Miikan or “living the right way” is based on the longstanding traditional and cultural knowledge of Anishinaabe people and includes a 90-day land-based treatment program. The purpose of this study was to explore and describe the lived experiences of participants in the Gwekwaadziwin Miikan land-based mental health and addiction treatment program during and after program participation by listening to their stories. In co-designing this study with participants, we recognized that Indigenous storytelling is a crucial way of sharing knowledge and history.

## Methods

We used an exploratory sequential mixed-methods design to describe participant experiences in the Gwekwaadziwin Miikan program. The study was co-designed with treatment program participants and based on input from the United Chiefs of Mnidoo Mnising (12 November 2019 meeting), the Elder Council (7 February 2020 meeting), and Knowledge Keepers (multiple meetings). We began with qualitative data collection and analysis to explore participant experiences and identify key themes, followed by descriptive quantitative analysis to contextualize program engagement and substance use outcomes. The findings were reviewed and approved by the Elder Council (at two meetings that took place on 15 August 2023 and 1 September 2023).

The project adhered to the Tri-Council Policy Statement: Ethical Conduct for Research Involving Humans (TCPS2) Chapter 9 titled “Research involving the First Nations, Inuit and Metis Peoples of Canada”, and OCAP® principles. Ethical approval was received from St. Michael’s Hospital in Unity Health Toronto.

Gwekwaadziwin Miikan participants may voluntarily engage in three phases of support on their journey to healing and wellness: 90 days on the land participating in circles with other participants and program staff, one-on-one meetings with program staff, and engagement in a range of activities including walks, canoe building, tool building, and meeting with Knowledge Keepers; a live-in aftercare program which incorporates traditional and cultural values of Anishnaabe people along with evidence-based Mental Health and Addiction therapeutic practices, education, and life skills for participants living in residence; and community aftercare (out-patient) which focuses on transition back into family and community by providing a client-centred spectrum of care utilizing community-based First Nation programs and Mental Health and Addiction services (*Home*, n.d.). Throughout the program, participants engage with the Redpath model that includes therapeutic sessions using traditional teachings and emotional and social competency skills as an alternative to the cognitive behavioural approach (Shaughnessy, n.d., 2001). While Gwekwaadziwin Miikan was originally designed to serve youth aged 19 to 30, the program has since expanded its eligibility criteria to include adults over the age of 30 in response to community need.

Data collection and analysis were performed by the research team with support from Gwekwaadziwin Miikan staff and Knowledge Keepers. One-on-one semi-structured interviews were conducted on the land by three research study team members (AS, AW, HW; research staff based in Toronto who are not Indigenous and who identify as settlers), and a Knowledge Keeper was usually present as requested by the participant. Site visits occurred on day 30 for cohort 1 (22 June 2021) and cohort 2 (12 October 2021), and on day 60 for cohort 3 (23 March 2022). During visits, the research team participated in circle ceremonies, ate meals with participants and staff, and stayed on-site. All participants participating in these three cohorts were invited to participate in the study. Follow-up interviews were conducted 6 months after the scheduled end of the 90-day land-based treatment program by telephone by one member of the research team (AS, AW, HW). Seven staff members were interviewed during the March 2022 visit to learn about their experiences with the program.

Interviews were conducted in English and audio recorded with consent. We used an interview guide that was pilot tested on five program participants and discussed with groups of program participants and the Council of Elders. A separate interview guide that focused on required training and challenges was used for staff. The results from all three visits were integrated and are reported together. We used a phenomenological approach to the qualitative analysis of interviews in order to summarize the experiences of participants and organize them into themes (Groenewald, 2004). A phenomenological approach was used as the study aimed to understand the complex experiences of participants. We (HW, AW, and AS) analysed the interview data in five steps: bracketing and phenomenological reduction, delineating units of meaning, clustering units of meaning to form themes, summarizing each interview, and summarizing themes and making a composite summary (Groenewald, 2004). We employed reflexivity throughout and validated findings by presenting them to other team members, participants, program staff, Knowledge Keepers, and members of the Elders Council. We analysed data cumulatively and stopped data collection when we determined that saturation was achieved after consulting with program staff, Knowledge Keepers, and members of the Elders Council.

Program completion rates and substance use at 6-month follow-up were measured through participant self-report during telephone interviews and summarized using descriptive statistics (frequencies and proportions) to provide contextual insight into the participants’ outcomes.

## Results

Over three land-based treatment cohorts, a total of 39 out of 40 (97.5%) participants agreed to participate in the study. All 14 participants in the first cohort, 11 of 12 in the second cohort, and all 14 participants in the third cohort participated. The characteristics of participants are summarized in Table 1.

**Table 1:** Participant characteristics (*N* = 39)

Characteristics	*n* (%)
Women, men	18 (46), 21 (54)
Age (years)	
18–30	24 (61)
31–40	8 (20)
40+	7 (18)

The experiences of participants during the land-based treatment program can be organized with four overarching themes that are discussed in detail below: connection with Indigeneity, connection with the land, trauma, and peer relationships. These themes are supported by Boxes 1, 2, and 3, which present representative quotes and thematic groupings to illustrate the connection between participant narratives and the analytic process.

**Table:** 

Box 1. Connection with Indigeneity
Reconnection:
*“I’ve learned how to introduce myself, little words here and there which is nice. And just the culture is in everything we do right, so its incorporated. I’m definitely getting back towards the spirituality that I once had like when my grandma was around.” P2*
*“I grew up on the reservation and I went to a very cultural school…So I know the tip of the iceberg of the culture, but this really drove me to realize that it’s not very systematic. It’s very open there’s no real wrong way of doing things- there’s different ways of doing things and I’m starting to realize that as long as you believe in the creator or your higher power that you’re not really doing anything wrong. And it’s given me a lot more knowledge of my culture. Because for so many years I moved away from home and I kind of just forgot about my background.” P16*
Working on the connection with culture had helped many participants in their mental health and addiction journeys. Being able to engage in cultural practices was said to be very beneficial.
*“You know, I’ve had some close contact with some elders in my past…but I discovered being here that doing all the things that I was once instructed to do but never did because I was always lost in my habits, here if I’m feeling something really intense and I don’t want to feel it or if I’m thinking of something really negative and I just want to escape from those thoughts, I’ve learned to kind of replace substance and alcohol with the medicines that we have at our disposal here. I’ll go and lay some sema in the fire and do a smudge and then I’ll go pick some cedar and make some cedar water and take a bath and I’ll drink some cedar tea and I’ll take a walk off the beaten path and just kind of get lost and sit down on the ground by a tree or on a rock and just kind of reground myself. And I’ve kind of just earned respect and appreciation for how all those things that my elders told me I need to start doing would actually work and I kind of wish I took that more seriously years ago because I’d probably be in a better place right now.” P12*
Learning about culture:
Wanting to know more and to connect was important for self-discovery.
*“…*I don’t know anything about my background like, I don’t know, some people here know their clan, how to speak their language, and I don’t know anything, right. It sucks. It feels like you don’t really have roots I guess, or you don’t really know your place, right. But I’ve learned more…” P5
Some participants discussed hoping to reconnect with themselves by participating in the land-based treatment program.
*“I wanted to get back in touch with myself and find myself again and I thought this would be the perfect place to do that…I was hoping to learn more about my culture and about myself” P6*
*“…I am trying to find who I am…well I know who I am, but I am trying to get back to who I want to be basically” P15*
*“Ever since I started drinking, it changed me. I lost my identity and myself. I lost my mindset and just lost myself” P1*

**Table:** 

Box 2.Connection with the land
*“So far the isolation [has been the most helpful part of the program]. Being out on the land really helps…being away from ‘friends’.”P8*
*“I feel for me, growing up I was really confused about my cultural identity. Um, and a lot of like the hardships that I’ve been through in life was through lack of direction, so I believe coming onto the land brings me back to that connection with mother earth and allows me the opportunity to learn more about cultural traditions and teachings…being connected to the land I think brings you back to a lot of honorabilities. Testing yourself physically which plays into the mental, your mental strength, emotional and spiritual…just getting back to the roots. I think there’s so many things we’re unconsciously aware of. So I think whether or not I address them, there’s so much work going on spiritually just by being connected back to mother earth.” P12*
*“…in any other recovery home or halfway house or treatment centre I’ve been in I was usually just a hop skip and a jump from a liquor store or trap house, so here I have to give 7 days notice to evac and I knew that if ever the going did get rough enough that I felt like escaping and relapsing, there would probably be a turning point within the 7 days between the time I give my notice and the time I would actually ship out that I’d probably change my mind.” P12*
Participants also spoke positively about land-based treatment as an alternative to other treatment settings like classrooms and clinics.
*“the reason I wanted to do the land based was because I didn’t wanna do none of the clinical stuff…you know sitting in the dead office with the dead lighting and you got all the questions coming at you and stuff.” P9*
*“…they sent me an email with a bunch of options for treatment, so I looked at all the websites and this was the only one that was land based. I’ve always felt better being outside. I’ve been homeless for 3 years um, and uh, I was staying in the forest…I figured coming here I could be outside, its not classroom based which I thought would be a bit boring and like, its just hard for me to sit still for so long…And like I said the culture thing is big for me. I thought that it would be more healing to be able to have that side of me be more fulfilled than just sitting in a classroom.” P5*

**Table:** 

Box 3. Relationships with peers
*“we all sit in circle and talk about our feelings and we all support each other so that’s a really big part of it too. We all talk about things together and once one of us shares then we all share and it gets really deep. And you let out all those feelings and all that pain and stuff that made you addicted to opioids or whatever the alcohol and stuff. So when you talk about it you feel a lot better. You may be upset for a little but after you’re done talking about it you feel amazing. So it’s healthy.” P12*

### Connection with Indigeneity

Participants spoke about connecting or reconnecting with Indigeneity as a reason for choosing land-based treatment. Some participants said they were not necessarily hoping to connect with their culture but were open to learning. Many participants said that part of reconnecting with their culture was remembering the knowledge that they had learned in the past but had since forgotten.

“growing up I used to go to [location] with my grandparents. Hunting, trapping, fishing, set up snares and whatever, and then I lost that when I started using substances…I lost touch. I’m trying to get it back.” P1

The effortlessness of recalling cultural information and practices during land-based treatment was expressed by multiple participants. Being present in an environment that fostered cultural connection helped participants to tap into the knowledge that they already possessed.

“…reconnecting, rebuilding…just being here, even the conversations or ceremonies, it’ll pop up, stuff that I do already know. It’ll help me remember it.” P27

Some participants were hoping to learn more about their culture because they had limited knowledge for various reasons. For instance, some participants did not learn about their culture while growing up.

“[I am hoping] to learn more because I grew up in foster care, so I didn’t grow up in a Native home. I grew up in all sorts of different homes.” P22

Strides towards reconnecting had been made by many participants at the time of the initial interviews after approximately 1 month of being on the land. Some participants expressed that they already felt more connected to their culture:

“[the program] has made me appreciate everything a lot more. I always wanted to gain more knowledge of my culture. It’s beautiful- we go for walks, we smudge and stuff like that. It was always something I wanted to gain more knowledge on, the language too. My dad is Ojibwe so I was hoping to gain a lot more knowledge on my culture and I’m getting that here. So it’s awesome.” P16

Most participants who discussed connection or reconnection spoke about how being on the land facilitated the connection and spoke of a connection to the land as well.

### Connection with the land

The land was a recurring theme throughout interviews. The land itself was a draw for many to the program and was repeatedly expressed as one of the biggest benefits of the land-based treatment program.

“I firmly believe that land-based treatment and reconnecting with the land and language as well is vital to, I guess my mental health… being on the land [has been the most helpful part]. Being by the water-the water is a big healer for me- taking walks in the bush, seeing the wildlife that’s out here as well is a big thing for me” P3

The land was discussed as being medicinal by participants as well, with some saying it could replace prescribed Western medicines for them. Referring to the land and wilderness environment, one participant said:

“…I’ve done sublicade and others and I’m here with nothing and I don’t even notice it you know, I don’t notice that I’m missing anything. Just this place itself is equivalent to methadone or suboxone.” P12

Participants said that there are pros and cons to both medical treatments and land-based treatment programs. Some participants felt that the land-based treatment program is better because it gets more to the “root of the problem” than other types of programs and particularly compared to substitution therapy such as methadone.

The remoteness of the land was discussed as a positive by many participants, particularly those who were enrolled in the summer when camp was on an island. Being physically removed had both practical implications, like limiting access to substances, and emotional implications.

“I’d say 97-99% of the time throughout my day I don’t even think about picking up a drink or a drug, whereas when I’m in another facility in a town or city where its readily available if I so choose to leave, I think about picking up at least once to 10 times a day. So here there’s a feeling of relief, you know, I don’t really have to focus on doing recovery work because I feel like the whole environment and the setting itself is doing half the work for me.” P12

The remoteness and disconnection were also a challenge for many. It was often talked about as a positive and a negative by the same participant. A lack of technology and the disconnection from family, especially from children, were discussed as some of the biggest challenges for participants, though many did acknowledge that this would be the case with other types of treatment programs as well. Some felt that while the down time was good for reflection, it felt like too much of a break.

### Trauma

Some participants talked about wanting to work through trauma, especially intergenerational trauma, as an important reason for deciding to participate in the land-based treatment program. One participant talked about experiencing racism in other treatment programs they had participated in in the past:

“This is my fifth treatment program…I was abstinent…I went in [year] to a treatment centre and then I relapsed after. There was a lot of conflict within AA, a lot of racism and stuff and I would talk about my spirituality and stuff and they’d be like nah nah nah if you don’t conform to the big book and this and that. There was a lot of talking about other people and a lot of stigma and stereotyping… I grew up in a family that was really traumatized by intergenerational trauma- a lot of sexual abuse a lot of violence…I didn’t feel in place because I felt like I was just gonna be looked at like I was different, like I was just a freak compared to their problems and then when I come here it’s like, it’s my people first of all. We’re all the same… I can set my mind straight and focus on and reflect on my negative behaviour patterns that I learnt along the way through my intergenerational trauma or my trauma…” P24

### Relationships with peers

Relationships with peers was a recurring theme that was discussed in different ways. Some participants talked about the large group setting and learning to interact with a group of peers as a challenge or source of anxiety. Some said this was only an issue when they first arrived, while a couple said it was one of the biggest challenges of the program for them generally.

“I was really anxious [when I first got here]. I struggle with social anxiety, so I struggled with that aspect. There were so many people.” P22

Peer relationships were also discussed by many as a positive aspect of their experience in the program. There was a lot of discussion around support and comradery and the feeling that the group was a family.

“meeting all the people here, like all the participants, it’s been really good. We’ve become a really tight family. We all say we love each other so that’s pretty tight. Shows how close we are”. P17

“we sit down at circle and we talk, we smudge around the fire, we’re all like a big family here and we let our feelings out and stuff like that and we do a Redpath program and it really brings out the roots and causes of your addiction. So when you talk about it and you cry and you feel those feelings you kind of let it out and feel a lot better after its done mentally and everything. So that helps a lot. To talk about stuff” P20

The group structure and learning to rely on one another were seen as a helpful part of the program by many.

“…the fundamental group based component of the daily outline, like doing a sacred fire, talking circle in the morning and the evening, always having a fire going under a sacred structure like an arbor, um you know, all of the sage and tobacco and sweetgrass and cedar I need, you know, the freedom to have time with myself with nature and also learn to rely on my peers. Uh, I think, the way that everybody has their roles and contributions that we kind of had to create on our own and find ourselves. Nobody, aside from weekly kitchen duties, nobody has really appointed us to any tasks so…there are some of us who make the fire and keep the fire which is kind of a traditional role, like the firekeepers, and then some of the girls are always out picking medicine and uh and me and a couple guys go out fishing when we can”. P12

One cohort had a mix of people over and under 30 and had more people in the cohort than the others. Some people in this cohort discussed the age gaps as a challenge and talked about a divide where the older people would stick together and the younger people would stick together. Some of the people under 30 felt as though the older people did not respect them as much due to their age and felt that they sometimes tried to tell them what to do. Some of the people over 30 talked about a lack of maturity in some of the younger people as a challenge that required patience. The program has since decided to separate these age groups in the future.

In addition to the themes discussed, at follow-up some participants stated that they wished to be more engaged in the Indigenous community but had not been able to yet due to COVID-19 restrictions. Many reported that they were working towards or had changed their goals, like maintaining or seeking out new employment or attending post-secondary school:

“I switched away from university…I got accepted into [college] for Indigenous wellness and addictions program. So then I might do social work or work with youth to prevent them from going down the path that I went down. I think college will be the best application.” P19

### Six-month follow-up

We conducted 6-month follow-up interviews with 20 out of 39 (51.2%) study participants. None of the 20 participants reported that they were using substances at the time of the 6-month follow-up interview. Five out of the 20 (25%) indicated that they had relapsed at some point since leaving the program but had since stopped using.

Most participants stated they were doing well mentally 6 months after the land-based treatment phase ended, though there were ups and downs. Some reported struggles with re-integration, for instance when going back home to a small town where past friendships involved substance use. Ongoing support through Gwekwaadziwin Miikan was discussed in follow-up interviews and was appreciated by participants, but one did find this to be a challenge since leaving the program:

“I felt real strong feeling of abandonment. You develop a relationship with the land-based counsellors and then all of a sudden its gone. It just stops. [Community aftercare counsellor] has been reaching out and its good but its not the same as the relationships you built over 3 months on the land.” P18

### Other findings

Participants also said that the land-based program is suitable for people who are ready to engage, that is, not in the thick of substance use. A few participants wished for more time designated to Redpath and found it to be the most helpful aspect of the program. Some participants also wished for more scheduled one-on-one time with counsellors rather than having to initiate one-on-one time themselves.

### Staff interviews

Seven staff were interviewed about their experience as Gwekwaadziwin Miikan staff members, and their experiences are summarized at the end of this section. When discussing reasons for becoming involved with Gwekwaadziwin Miikan, staff mentioned growing up in an Indigenous family and wanting to share culture or knowledge with program participants and give back to the community; growing up separated from their Indigenous culture and the land and wanting to reconnect and learn more about Indigenous roots; past experiences with outdoor and land-based activities (e.g., canoe instructor, camp counsellor) and wanting to work on the land; past experience with mental health and addiction; being referred to the program by friends or family; and growing up in a small community on Manitoulin Island and being referred internally by existing program staff. The staff interviewed suggested some changes, like increased mandatory training in mental health and addiction counselling and increased training in camp-related and outdoor skills (e.g., working a wood stove). Overall, the staff members reported positive experiences working with the program.

## Interpretation

This mixed-methods study found that a land-based treatment program for Indigenous people with mental health and substance use challenges promoted connections to Indigeneity and to the land. Connecting to the land was identified as one of the main goals many participants were hoping to achieve through the land-based treatment program, and in follow-up interviews, many said this was achieved. The qualitative and quantitative findings were used in parallel to provide a fuller understanding of the program’s impact. While qualitative data captured participants’ experiences and perceived effects of the program, quantitative data contextualized these findings by summarizing program completion and substance use at follow-up. These findings align with half of participants who completed follow-up interviews 6 months after completing the program reporting they were no longer using substances.

Our findings are in line with previous research that suggests a sense of connection to the land is an essential part of Indigenous well-being, and that mental health interventions centred around connection to land are appropriate for use in Indigenous communities (Ahmed et al., 2023b; Walsh et al., 2020). Previous studies have shown that land-based subsistence and ceremonial activities positively influence community engagement as well as spiritual, physical, emotional, and mental health of Indigenous adults (Ahmed et al., 2021; Walsh et al., 2020). In this study, at the time of follow-up, some participants noted that they intended to become more engaged in their community and the majority discussed improvements to spiritual, emotional, and mental health by way of connection to Indigeneity and the land. Gwekwaadziwin Miikan operates in remote settings, but land-based treatment has been shown to be beneficial for Indigenous youth in urban settings as well (Hatala et al., 2020). Healing through culture is an effective model for addictions treatment (Dobson & Brazzoni, 2018).

An uncontrolled observational study cannot demonstrate causation, and the follow-up rate was low. Interviews took place in the presence of Knowledge Keepers who supported the program, and this may have affected participant responses. The researchers analysing the data were not Indigenous or experts in Indigenous health, which may have influenced data collection and analysis. Findings were discussed with Elders and Knowledge Keepers to validate and contextualize our interpretations. This mixed-methods study of the experiences of participants in a land-based treatment program for mental health and addictions provides detailed insights based on substantial data and provides insights that could be used to inform land-based treatment programs.

## Conclusion

A land-based treatment program for Indigenous youth and adults with mental health and addictions challenges provided connections to Indigeneity and to the land, and the participants who completed a follow-up interview were not using substances. Land-based treatment programs could be implemented to mitigate the harms of colonization in conjunction with or in addition to other supports including opioid agonist substitution therapy. Indigenous people should be offered land-based treatment programs, and timely access to treatment can be facilitated by supporting existing and new programs in addition to returning land to Indigenous people as part of decolonization.

## Contributions to knowledge

What does this study add to existing knowledge?

- This study provides detailed insights into the experiences of participants in a land-based treatment program for Indigenous people with mental health and substance use challenges. Our findings highlight the importance of connection with Indigeneity and the land in promoting health and well-being. Additionally, the study underscores the significance of offering culturally relevant alternatives to conventional treatment methods, supporting the broader implementation of land-based and culturally centred healing practices.

What are the key implications for public health interventions, practice, or policy?

- Indigenous people should have access to land-based treatment programs. This could help promote health generally and especially for people with mental health and substance use challenges.

## Supplementary Information

Below is the link to the electronic supplementary material.

Supplementary file1 (66.8 KB)

## Data Availability

The data that support the findings of this study are available from the corresponding author (NP), upon reasonable request.

## Code availability

N/A.
