# Supplementary material for: Experiences of land-based treatment program for Indigenous people: a mixed-methods study of Gwekwaadziwin Miikan
Source: Can J Public Health. 2026 Feb 9;117(5):964–72. doi: 10.17269/s41997-025-01131-4 (PMC13680600; doi:10.17269/s41997-025-01131-4)
Supplement: Supplementary file 1 [file 41997_2025_1131_MOESM1_ESM.docx]

**MAANDOBIIGAN**

*Nenda gashkichigaadeg:* Akiingh enji temigag noojimowin nankiiwinan newen inendamowin mina yaawin miinwaa omamaanjimigowin maanendaagwog gnimaa adaa aazhidesenon newen myaanaadog madookidaagewinan owi oshki digoshinajig zhichigewinan gewe Giizhaa Agaa Yaajig bemaadizijig owi naanaagwisidoongh Anishinaabe inaadiziwinan miinwaa ezhi nakweshkigaadeg owi noojimowin e’dikobidemigag owi aki. Owi enaabidag maanda gnigsijigaademigag nikiiyaa zhichigewin nda-gikenjigewin agii aawon awii dibaachigaademigag newen gikendamowinan gewe waaji ayaajig zhiwe owi Gwekwaadziwin Miikan akiingh enji temigag inendamowin mino yaawin miinwaa omamaanjimigowin noojimowin nankiiwin.

*Zhichigewinan:* Owi gnigsijigaademigag nikiiyaa zhichigewin nda-gikenjigewin agii maamowi mazininigaademigad gewe nojimowin nankiiwin waaji ayaajig miinwaa onji baamigad owi inendamowiniwaa gewe Maamowi Yaajig Gimaag owi Mnidoo Mnising miinwaa owi Gechi Piitizijig Giigido(ninwog/kwewog) miinwaa Gikendaasowin Ginowendamajig. Ozhisijigaadeg digo ayaad bemaadizid bebezhig gagwedwewin agii zhichigaadenon epiichi temigag owi 90 giizhigag akiingh enji temigag noojimowin nankiiwin miinwaa nigodwaaswi giizis ishkwaasemigag owi akiingh enji temigag noojimowin.

*Makigaadegin:* Ngii giishtonaanin 39 bebezhing bemaadizijig gagwejimigaaziwaad epiichi noojimowaad miinwaa 20 nigodwaaswi-giizis aanji gagwejimigaaziwaad zhiwe giigido biiwaabikoonsingh miinwaa age ayaangh gaataadibiwinan miinwaa bkaan maamowi yaangh dinakimigiziwinan. Niiwin aazhiwi-semigag debaachigaademigag agii waawiinjigaadenon: dikobizowin owi Giizhaa Agaa Yaajig Bimaadiziwin, dikobizowin owi aki, wiisigendamowin, miinwaa wiiji piitiziimaaajig wiijkiwendiwin. Kina 20 waaji ayaajig gashkitowaad owi aanji gagwejimigaaziwaad agii wiindamaagewog gaawii agii nikaazisiinaa-aa aaboon apii 6 giizis. Naanon (25%) agii wiindamaagewog azhesewin owi apii shkwaa maajaawaad owi nankiiwin dash gimaawipii agii boonitaawog nikaaziwaad.

*Giizhaajimowin:* Owi Gwekwaadiziwin Miikan akiingh enji temigag noojimowin nankiiwin agii miigwemigad dikobizowin owi Giizhaa Agaa Yaaji bimaadiziwin miinwaa owi aki gnimaa age aazhidetomigag zhichigewinan owi naajitowaad Giizhaa Agaa Yaajig bemaadizijig owi ado kiimiwaan. Ninda makigaadewinan adaa naadmaagemigad aasgaabiwichigaadeg owi woshme maajiishkachigaadeg akiingh enji temigag noojimowin nankiiwin miinwaa zhaazhowaach dnowaa nankiiwinan owi naanaagwisingh Giizhaa Agaa Yaajig bemaadizijig gikendamowinan miinwaa noojimowin zhichigewinan.

Ishpi Kidiwinan: Giizhaa Agaa Yaajig bemaadizijig; Inendamowin mina yaawin zhichigewin; Maziwe Wiiyaw mina yaawin; Nakiiwin dibizhigaadeg

**MOOKAAJMOWIN**

Makigaadeg mashkoziiwin onji dikobizaowinan owi aki aawon gnimaa nakwetamowin owi zhichigewin owi oshki digoshinajig owi aakwaaji bkebijigaaziwaad Giizhaa Agaa Yaajig bemaadizijig ado kiiman. Ahmed et al., 2021, 2023a, 2023b; National Inquiry into Missing and Murdered Indigenous Women and Girls (Canada) et al., 2019). Eshi waasesemigag zhiwe bezhig waaji ayaad ekidad, “…*aanji dikobizaaan, aanji zhito-aan…goweta ayaa-aan zhinda, boochigo newen dinaangidonwinan maage manitokewinan, ada moogsemigag, gego zhaazhigwa gekendamaa. Nga naadmaagwon awill makwendamaa owi”.* Akiingh enji temigag noojimowin nankiiwinan adaa aanji dikobinigemigad Giizhaa Agaa Yaajig bemaadizijig owi aki aawong waajibidemigag owi noojimowin zhichigewin (Mattick et al., 2014) miinwaa nakwetaan aanke bimaadizijig wiisigendamowin, maage wiisigendamowin agaa zhiwebiziwaad Giizhaa Agaa Yaajig bemaadizijig maanzhi doodamowin gikendamowin gewe aakwaadog oshki digoshinajig zhichigewinan dibishko gonaa endinizingh gikinomaage gamgoon. (Gittings et al., 2024). Akiingh enji temigag noojimowin nankiiwinan agii waawiinjigaadenon owi “…mino yaawin maage noojimowin nankiiwin maage zhichigewin etemigag zhiwe zaagiji gichi odena, ngoji apii odenaangh maage bigod’kamig ngoji zhiwe akiingh owi onjida jichaag gitigaadeg awii gagwekwendaagwog owi aki gichi inenjigaadeg miinwaa minaadenjigaadeg. Owi aki n’saschigaademigad aawongh owi bimaadiziimigag miinwaa wiijiindiwin gewe bemaadizijig nakiiwaad zhiwe nojimowin zhichigewin.”(Redvers, 2016). Owi zhichigewin nda gikenjigaadeg newen zhaangswi akiingh enji temigag noojimowin nankiiwinan digosingh wiiyaw nakiichigaadeg agii giikinaan wiiyaw mino yaawin, jichaag mino yaawin, inendamowin mino yaawin miinwaa odeniwingh gikobizowinan ondinigaademigag newen dibaabaajigewin bebezhig dibaamjigaadeg newen gagwejimaagewinan miinwaa bezhig genwaabmdamijig maamowi yaajig. (Ahmed et al., 2021). Dikobizowinan owi bigwaajiwiingh adaa nanaaitoomigad inendamowin mino yaawin (Nejade et al., n.d.) miinwaa gichi ishpenjigaadeg gewe Giizhaa Agaa Yaajig bemaadizijig (Ahmed et al., 2021; Cunsolo Willox et al., 2012).

Gashkitoongh awii misenjigaadeg noojimowinan onji nibewaaboon penimanjigaadeg dibasaamigad megwaa owi zaam niibna mashkikiinin mazinigaansan owi miigwengh nibewaaboon mashkikiin miinwaa owi sweshkaamigag debwesinag wiindamaagewin newen onji miigwemigad owi gichi maajiishkaamigag owi minik newen nibewaaboon niwendaagwog wiisigikaagewinan apii owi 1990s (Van Zee, 2009). Mino Yaawin naagidowenjigewin temigag gnimaa adaa aakwaadod miinwaa niizaanod gewe Giizhaa Agaa Yaajig bemaadizijig, memdage gwa apii n’dowaabmdamiwaad naagidowenjigewin newen inendamowin mino yaawin miinwaa aaboon nikaaziwin maanendaagwog (Ahmed et al., 2021, 2023a; Morton Ninomiya et al., 2022; Nelson & Wilson, 2017). Akiingh enji temigag noojimowin nankiiwin adaa aankesinon aanin bkaan biminizhigaademigag wenaamjiganan onji indendamowin mina yaawin namjiwinan miinwaa aaboon nikaazangh temigag noondaaj nakiimigag miinwaa temigag (Klimas et al., 2018). Mashkiki nikaaziwin aabiziichiganan onji ishkode-aabo miinwaa nibewaaboon nikaazang aakoziwin naadawisemigadoon aanin ezhiwebak dibishko gonaa aabiziichigan majimsingh miinwaa owi minik giizhigadoon owi niibna aaboon nikaaziwin dash gaawii gwa gegeti naazhisesino aaboon nikaaziwin owi gnesh minik (Jonas et al., 2014; Mattick et al., 2014). Miinwaa aanke miigwengh mashkiki nikaaziwin aabiziichiganan gewe Giizhaa Agaa Yaajig bemaadizijig agaa maanaaji doodaagiwaad owi nibewaaboon maanzhisewin, owi adaa eshaawaadendaagwod awii temigag misenjigaadeg owi akiingh enji temigag noojimowin nankiiwinan. Mashkikiinini/kwe nyaagidowenimaajig Giizhaa Agaa Yaajig bemaadizijig gewe gnimaa adaa misendaanaa-aa owi miingaaziwaad akiingh enji temigag noojimowin nankiiwinan gnimaa gaawii adaa gikendiziinaa-aa newen akiingh enji temigag noojimowinan aawechigaadeg newen bkaan noojimowin dnowaan.

Owi Gwekwaadziwin Miikan Inendamowin Mino Yaawin miinwaa Mamaanjigowin Nankiiwin aawon owi wiiji nakiindiwin biitwi nigodwaaswi Inakaaneziwinan, owi Manidoo Minis endaajig miinwaa owi Gimaawin maampii Ontario. Gwekwaadiziwin Miikan maage “bimaadizingh gagwek nikiiyaa” ozhisijigaademigad owi gichi gnesh epiichi temigag gete bemaadiziwin miinwaa inaadiziwin gikendamowin gewe Anishinaabe bimaadizijig miinwaa digosingh owi zhaangswi giizhigad akiingh enji temigag noojimowin nankiiwin. Owi enaabidag maanda nda gikenjigewin aawon awii ginowaabmjigaadeg miinwaa dibaatomongh owi gikendamowin gewe digo yaajig zhiwe owi Gwekwaadziwin Miikan akiingh enji temigag inendamowin mino yaawin miinwaa mamaanjiminigowin noojimowin nankiiwin epiichi miinwaa shkwaa nankiiwin digo yaawin owi bizinjigaadeg ado dibaajimowinan. Owi wiiji mazininigaadeg maanda nda gikenjigewin gewe digo yaajig, ngii nisidiwinaanaa owi Giizhaa Agaa Yaajig bemaadizijig dibaajimowin aawon nendowendaagwog nikiiyaa owi maadokiingh gikendamowin miinwaa mewizha agaa zhiwebak.

**ZHICHIGEWINAN**

Ngii nikaazinaanin owi nanaadawi gikendamaangh aankesingh gnigsijigaadeg zhichigewinan mazininigaadeg awii dibaachigaadeg digo yaajig gikendamowiniwaa zhiwe owi Gwekwaadziwin Miikan nankiiwin. Owi nda gikenjigewin agii digo-mazininigaademigad gewe noojimowin nankiiwin digo-yaajig miinwaa onjibaamigag inendamowiniwaa gewe Maamowi Yaajig Gimaag owi Mnidoo Mnising (midaaswi niish Baashkaakodin-giizis 2019 nakweshkidaadiwin), miinwaa owi Gechi Piitizijig Giikimigejig (niizhwaaswi Mkwa-giizis 2020 nakweshkidaadiwin) miinwaa Gikendaasowin Genowendamajig (niibna nakweshkidaadiwinan). Ngii onji maajitaami owi dibaabajigewin maandonigewin miinwaa dibaabiishkojigaadeg awii ginowaabmjigaadeg digo-ayaajig gikendamowinwaa miinwaa gikendaagwod gechi piitendaagokin, aanikesingh owi dibaachigaadeg dibaginjigewin dibaabiishkojigaadeg awii nisadawendaagwog nankiiwin wiikijinakiingh miinwaa aaboon nikaazang ezhiwebak. Newen makigewinan agii nda-gikenjigaadenon miinwaa minwaabmjigaadenon gewe Gechi Piitizijig Giikimigejig (odi niish nakweshkdaadiwinan agaa temigag 15^th^ Manoominike-giizis 2023 miinwaa 1^st^ Waababagaa-giizis 2023).

Owi anookaajigan agii naagidoon owi Niswi-giigidowinag Naaknigewin Wiindamaagewin: Ethical Conduct for Research Involving Humans (TCPS2) Chapter 9 titled “Research involving the First Nations, Inuit and Metis Peoples of Canada”, and OCAP® principles. Naagijigewin minwaabmjigaadeg agii debinigaademigad onji St. Michael’s Akoozii-gamig zhiwe Unity Health Toronto.

Gwekaadiziwin Miikan digo yaajig adaa inendamog awii wiiji ayaawaad newen niswi bimisemigag owi aasgaabiwichigewin ado bimiyaawaad owi nojimowin miinwaa mino bimaadiziwin. Zhaanghswi giizhigad zhiwe aki wiidokidaazong zhiwe gaataadibiwinan owi gewe bkaan digo yaajig miinwaa nankiiwin enkiitaagejig, bebezhig nakweshkidaadiwin gewe nankiiwin enkiitaagejig, miinwaa wiiji ayaangh newen nowonj dinakimigiziwinan, e’digosingh bibaamsengh, jiimaan zhichigaadeg, nikaaziwinan zhitoongh, miinwaa nakweshkowindwaa gewe Gikendamowin Genowendamajig; dinizingh zhiwe shkwaa ginowenjigewin nankiiwin owi e’digosingh Anishinaabe zhichigewin miinwaa inaadiziwin naagijigewinan gewe Anishinaabe bemaadizijig digobidemigag age gikenjigaadeg-onjibaamigag Inendamowin Mino Yaawin miinwaa Mamaanjimingowin zhichigewinan, gikinomaadiwin, miinwaa bimaadizingh nikaaziwinan nigajitowinan gewe digo yaajig endaajig zhiwe wiigwaamingh; miinwaa odenwingh aabaji ginowenjigewin (ngoji bkaan endaad) owi memdage ginowaabmjigaadeg owi biskaabiingh owi inoodewiziwin miinwaa odena owi miigwengh bemaadizid-bebezhig nowoonj dnowaa ginowenjigewin nikaazong endinakiingh temigag Inakaaneziwn nankiiwinan miiinwaa Inendamowin Mino Yaawin miinwaa Mamaanjimigowin zhichigewinan (Home, n.d.). Maziwe gwa owi nankiiwin, digo yaajig wiijiyaawog owi Redpath gikinowaabanjigon owi digosingh aabiziichigan zhichigewinan nikaazong gete dibaajimowinan miinwaa izhayaawin miinwaa wiijikiwendiwin gashkichigaadeg nigaajiiwinan ayaawong owi meshkod owi inendamowin izhiwebiziwin nikaazongh (Shaughnessy, n.d., 2001). Apiichi Gwekwaadziwin Miikan ntam agii zhisijigaademigad awii nakiitwindwaa eshkiniigijig epiitizijig 19 apiinish 30, owi nankiiwin agii ani giikinigaademigad owi miigsewin nendowendaagwog awii digobizowaad netaawigijig woshme epiitizijig 30 owi nakwetamong owi odenaangh menezingh.

Dibaginjigewin maandonigaadeg miinwaa dibaabiishkojigaadeg agii nakiiwog gewe nda-gikenjigewin enkiijig owi aasgaabwichigewin onji Gwekwaadziwin Miikan enkiitaagejig miinwaa Gikendaasowin Genowendamjig. Bebezhig bemaadizijig zhisijigaadeg gagwedwewinan agii ozhichigaadenon zhiwe akiingh gewe niswi nda-gikenjigewin waaji nakiindijig (AS, AW, HW; nda gikenjigewin enkiitaagejig odi Toronto gaawii Gizhaa Agaa Yaajig ayaawisijig miinwaa eshi waawiindiziwaad agaa gashki digoshinajig) miinwaa Gikendaasowin Genowenjiged gagwetch agii digobi ezhi gagwedwed owa digo ayaad. Azhaangh akiing enji temigag agii tenoon apii owi giizhigad 30 owa Naasaab ezhiwebiziwaad 1 (22 Odemin-giizis 2021) miinwaa Naasaab ezhiwebiziwaad 2 (12 Binaakwe-giizis 2021), miinwaa owi giizhigad 60 owa Naasaab Ezhiwebiziwaad 3 (23 Ziisbaakdoke-giizis 2022). Epiichi nibwaachewaad, owi nda-gikenjige waaji nakiindijig agii wiidokaaziwog zhiwe gaataadibingh manidokewinan, wiidopimaa’aan gewe digo ayaajig miinwaa agii diniziwog zhiwe akiingh. Kina digo yaajig wiiji ayaawaad ninda niswi naasaab ezhiwebiziwaad agii wiikimigaaziwog awii digokaaziwaad zhiwe nda-gikenjigewin. Aanji gagwedwewinan agii tenoon nigodwaaswi giizis shkwaa inaakinigaadeg ekwaasemigag 90 giizhigadoon aki enji temigag noojimowin nankiiwin zhiwe giigido-biiwaabikonsingh owa bezhig waajiiwed owi nda gikenjigewin waajinakiindijig (AS, AW, HW). Niizhwaaswi enkiitaagejig agii gagwejimigaaziwog epiichi owi Ziisbaakdoke-giizis 2022 nibwaachewin awii gikendamowaad owi ado gikendamowin owi nankiiwin.

Gagwedwewinan agii dinaangidonam owi Zhaagnaashiimiwin miinwaa dibaajimowin agii biindiwewesijigaademigad owi inendamowaad. Ngii nikaazinaa owi gagwedwewin gikinowaamjigan owi agaa oshki gajichigaademigag gewe naanon nankiiwin digo yaajig miinwaa agii dazhinjigaademigad gewe maamowi yaajig owi nankiiwin digo yaajig miinwaa owi Giikimigejig gewe Gechi Piitizijig. Owi bkaan gagwedwewin gikinowaamjigan owi mayaa awi nendowendaagwog gikinomaadiwin miinwaa maanendamowinan agii nikaazigaadenon gewe enkiitaagejig. Newen makigewinan onji kina niswi nibwaachiwewinan agii digosijigaadenon miinwaa wiindamaagem maamowisingh. Ngii nikaazinaa owi gagwek ayaawong debwemigag zhichigewin owi dibaabajigewin dibaabiishkojigaade newen gagwedwewinan owi awii dikobiimaangh newen gikendamowiniwaa gewe digo ayaajig miinwaa ozhisido-aangh newen zhiwe debaachigaadeg (Groenewald, 2004). Owi gagwek ayaawong debwemigag zhichigewin agii nikaazom awii nisastamongh newen gwiiniwi dnowaa gikendamowiniwaa gewe digo ayaajig. Niinwi (HW, AW, miinwaa AS) ngii dibaabiishkodaanaa owi gagwedwewin dibaginjigewin zhiwe niiwin nikiiyaa: maamiwisidoongh miinwaa gagwek ayaayong debwemigag naazhinigaadeg, gikinwaasijigaadeg bayezhigwang edimoomigag, digosidoongh bayezhigwang owi edamoomigag awii zhizidoongh ayaawong, dikobiigaadeg bebezhig gagwedwewin, miinwaa dikobiigaadeg ayaawong miinwaa zhichigaadeg ayaawong maamowibiigaadeg (Groenewald, 2004). Ngii nikaazinaa basabaagag gabewi miinwaa debwemigag makigaadegin owi miigwe-aangh gewe bkaan waaji nakiimdwaa, digo ayaajig, enkiitaagejig Gikendamowin Genowendamajig miinwaa eyaajig owi Gechi Piitizijig Giikimigejig. Ngii dibaabiishkodonaa dibagenjigewin ombishkaamigag miinwaa ngii nigaasidonaa dibaginjigewin apii agii owi moshkin agii gikendaagwog gashkichigaadeg shkwaa ginoojigaaziwaad enkiitaagejig, Gikendamowin Genowendamajig miinwaa eyaajig owi Gechi Piitizijig Giikimigejig

Nankiiwin giizhiitaangh minik miinwaa aaboon nikaazongh owi nigwadwaaswi giizis aanji ginowaabmjigaadeg agii dibizhigaadenon nikaazong digo ayaad dibaajimad epiichi giigido-biiwakoonsingh gagwejimigaaziwaad miinwaa maandobiigaadeg nikaazong nisidiwinaagwog ginjigaadegin (wewiiba miinwaa minik) awii miigwengh ezhiwebak waabmjigaadeg zhiwe owi digo ayaajig maajiishkaawin.

**MAKIGAADEG**

Woshme niswi akiingh enji temigag noojimowin naasaab ezhiwebiziwaad, owi kina minik gewe 39 owi 40 (97.5%) digo syaajig agii nendamog awii wiijii’ewaad zhiwe nda-gikenjigewin. Kina 14 digo ayaajig zhiwe owi ntam naasaab ezhiwebiziwaad, 11 gewe 12 zhiwe ekwa niizhingh naasaab ezhiwebiziwaad miinwaa 14 digo ayaajig zhiwe owi niswi naasaab ezhiwebiziwaad agii wiiji-yaawog. Newen izhi-ayaawaad gewe digo ayaajig maandobiigaademigad zhiwe Naaksijigan 1.

Newen gikendamowiniwaa digo ayaajig epiichi owi akiingh enji temigag noojimowin nankiiwin adaa zhisijigaademigad owi niiwin aazhiwi-semigag debaachigaademigag agii waawiinjigaadenon maampii nisawiingh: dikobizowin owi Giizhaa Agaa Yaaji Bimaadiziwin, dikobizowin owi aki, wiisigendamowin, miinwaa wiiji piitiziindiwin wiiji kiwendiwin Niswi debaachigaademigag aasgaabiwichigaadenon newen makakoonsan bezhig apiinish niswi, owi maagwemigag naabinootawaabiiganan miinwaa debaachigaadeg maamwisijigaadeg awii waabmdowengh owi dikobijigan biitwi digo ayaad agaa kidiwaad miinwaa dibaabiishkojigengh nikiiyaa.

*Dikobizowin owi Giizhaa Agaa Yaajig Bimaadiziwin*

Digo ayaajig agii dibaataanaa-aa owi dikobizowaad miinwaa aanji dikobizowaad owi Giizhaa Agaa Yaajig Bimaadiziwin ayaawong enji wenaamdamiwaad akiing enji temigag noojimowin nankiiwin. Aanin digo ayaajig agii kidwog gaawii gwa gegeti bigosendamasiiwog awii dikobizowaad ado inaadiziwin dash agii inendamoog awii gikendamowaad. Niibna digo ayaajig agii kidwog owi ayaawong owi anji dikobiziwaad ado inaadiziwin agii aawon makwendamowaad owi gikendamowin agaa gikendamowaad aazhigwa dash agii nendaanaa-aa.

*“ombigyaan ngii azhaa odi [ngoji] wiijiiwog ndo gikaawinag. Giiwise-aan, nsojigeyaan, giigokeyaan, zhisido’aan nisojiganan miinwaa wegwen gwa, amiidash agii naajito-aan owi apii agii majitaayaan nikaaziyaan aaboon….ngii wanishin. Ndo gajitoon ne-aab awii debinamaa.” P1*

Owi wenipanag owi makwendamowaad inaadiziwin gikendamowin miinwaa zhichigewinan epiichi temigag akiing enji temigag noojimowin nankiiwin agii dibaataanaa-aa gmaaminig gewe digo ayaajig. Ayaangh zhiwe owi zhichigewin gagaandinigaadeg inaadiziwin dikobizowin agii naadimaagaaziwog digo ayaajig awii nikaaziwaad owi gikendamowin aazhigwa agaa yaamiwaad.

*“…aanji dikobizayaan, aanji maajiigiyaan…goweta ayaa-aan maampii, awaa’aaj gwa ganoondiwin maage manidokewinan, nga makwendaan, newen zhaazhigwa agaa gikendamaa. Nga naadmaagwon awill makwendamaa owi”. P27*

Aanin digo ayaajig agii bigosendamog awii gikendamowaad woshme ado inaadiziwin zaam agii ayaanaa-aa nondesimigag gikendamowin owi nowonj onji. Dibishko gonaa, aanin digo ayaajig gaawii agii gikendiziinaa-aa ado inaadiziwin apii ombigwaad.

*“Ndo [bigosendam] awii gikendamaa woshme zaam ngii koginigwo zhiwe gimaa biminigowin, dash gaawii ngi koginigosii owi Anishinaabe endaangh. Ngii ombig nowonj dnoowaa bkaan endaangh.” P22*

Maajiishkaawin owi aanji dikobiziwin agii zhitonaa-aa niibna digo ayaajig apii oshkintam gagwejimindwaa megwaach gwa nigo giizis apii ayaangh aki. Aanin digo ayaajig agii wiindamaagewog aazhigwo agii izhayaawog woshme dikobizowaad ado inaadiziniwaa:

*“[owi nankiiwin] maaji maamiikwendaan kina gego woshme gichi niibna. Ngii aabaji dowendaan awii debinamaa woshme gikendaasowin owi ndo inaadiziwin. Ginaajiwon- ndo bibaamsemi, ndo bakwenezigemi miinwaa nowonj owi dnowaa. Pane gwa agii aawon agaa dowendamaa awii debinamaa woshme gikendamowin, owi iniwewin age. Noos aawi Ojibwe dash ndo bigosendam awii debinamaa woshme gichi niibna owi ndo inaadiziwin miinwaa ndo ani debinaan owi maampii. Dash maamiikwendaagwod.” P16*

Gegaa kina digo ayaajig agii dazhindaanaa-aa dikobiziwin maage aanji dikobiziwin agii dibaataanaa-aa owi ayaangh zhiwe akiing agii naadmaagemigad owi dikobiziwin miinwaa agii dibaataanaa-aa owi dikobizowin owi aki age.

*Dikobizowin owi aki*

Owi aki agii aanji dibaachigaademigad zhiwe gagwejimigowin. Owi aki agii aawon wiikibijigewin gewe niibna owi nankiiwin miinwaa agii aanke dibaajimowog aawongh owi gichi bamitaagewin owi akiingh enji temigag noojimowin nankiiwin.

*“Ndo gagetin debwewendam owi aki enji temigag noojimowin miinwaa aanji dikobiziwin owi aki miinwaa iniwewen age gichi dowendaagwog, ndo inendam ndo inendamowin mino yaawin… ayaa-aan zhiwe aki [agii aawon owi memaanji naadmaagemigag]. Ayaa-aan jiigibiig-owi nibi aawon gichi naandowechigan owi niin- bibaamseyaan mitigwaaki, waabmdamaa bigoji bimaadiziwin etemigag zhinda gichi aawon onji niin” P3*

Owi aki agii dazhinjigaademigad aawongh mashkikiiwong gewe digo ayaajig age, owi aanin kidwaad adaa meshkodsin maagwengh zhaagnaashii mashkiki wiinwaa onji. Dibaatamowin owi aki miinwaa bigodwaaki ayaangh, bezhig digo ayaad agii kida:

*“… Ngii nikaazan mashkiki maagaadong nikaazang aaboon miinwaa bkaan gego miinwaa maampii ndo ayaa gaawii gego ayaanziwaa miinwaa gaawii ndo maaminaamdaziin, gaawii ndo maaminaamdaziin owi mesinamaa gego. Zhinda maampii goweta dibishko aawon owi mashkikiin methadone maage suboxone maagaadong mamaanjigowin.” P12*

Digo ayaajig agii kidwog owi tenoon nishing miinwaa gaawi nishizinag neniish gwa mashkiki noojimowinan miinwaa akiing enji temigag nojimowin nankiiwinan. Aanin digo ayaajig agii zhayaawog owi akiing enji temigag nojimowin nankiiwin woshme nishin zaam woshme zhaamigad owi “enji maajitaamigag zinagiziwin” dash newen bkaan dnowaa nankiiwinan memdage gwa aawechigaadeg owi meshkwod naandwechigewin dibishko gonaa nibe-waabo.

Owi bigodwaaki owi aki agii dazhinjigaademigad aawongh e’nishingh gewe niibna waaji ayaajig, memdage gwa gewe agaa bozigebiigewaad apii niibingh apii gabeshiigamig agii temigag minisingh. Ayaangh ngoji bkaan agii tenoon niish endodaagemigag, dibishko naazhinigaadeg misendamong nibewaaboon, miinwaa ishayaangh dodamowin.

*“Ndo akid 97-99% owi minik epiich giizhigag gaawii awaa-aaj ndo inendizii owi ombinimaa minikwewin maage maji mashkiki, dash owi apii ayaa-aan bkaan naandwechige gamig zhiwe odena maage gichi odena zhiwe wenpanag awii debinamong giishpin wenaamdamaa awii zaagijiitimaa, ndo inendaman owi ombinimaa gnigen gwa nigodingh maage midaachingh ensa giizhigad. Dash maampii temigad owi izhayaangh naangendamowin, agii gikendaa ana, gaawii memkaach nda naagidowaabmdaziin owi nakiiyaan noojimowin nakiiwin zaam ndo inendam owi gaatiiwiingh miinwaa zhiwe enji temigag zhichigemigad owi aabitaa nakiiwin niin onji.” P12*

Owi bigojiiwong miinwaa dikobizasingh age agii aawon maanendaagwog. Agii minwe-aapii agii dibaachigaademigad owi aawongh minowii miinwaa maanaajiwii owa naasaab ayaawid digo ayaad. Owi tesinag waasamowin nikaaziwinan miinwaa dikobizasingh gewe inoodewizin, memdage gwa binoojiinig, agii dazhinjigaadenon aawinon aanin memaanji maanendaagwog gewe digo ayaajig, aanawi gwa niibna agii nisastaanaa-aa maanda adaa aawon newen bkaan dnowaa noojimowin nankiiwinan age. Aanin agii nendamog owi apii bizaaniyaangh agii nishin awii naanaagidowendamongh, agii nendaagwod zaam niibna kowe nwebingh.

*Wiisgendamowin*

Aanin digo ayaajig agii dibaataanaa-aa owi dowendamowaad awii nakiitamowaad wiisgendamowin, memdage gwa aanke bimaadiziwin wiisgendamowin, aawong gechi piitendaagwog enji giizhendamong awii digo ayaangh awii wiiji ayaangh zhiwe akiingh enji temigag noojimowin nankiiwin. Bezhig owa digo ayaad agii dibaataan agii gikendong bkaan enjigewin zhiwe bkaan noojimowin nankiiwinan agaa wiiji ayaad agaa bimisemigag.

*“Manda aawon ndo naanwi noojimowin nankiiwin…Ngii booniikaanan…Ngii azhaa [biboon] odi noojimowin wiigwaam miidash miinwaa agii azheseyaan agaa shkwaa temigag. Agii baatiinad miigaadsewin zhiwe AA, niibna bkaan enjigewin miinwaa nowonj gego miinwaa ngii dinaangidoon dibaatamaa ndo jichag miinwaa nowonj gego miidash agii aawiwog dibishko nah nah nah giishpin bwaa naagidowin owi gichi mazinigan miinwaa maanda miinwaa owi. Agii temigad owi niibna dibaajimigaaziwaad bkaan bemaadizijig miinwaa niibna maanaaji waawinigowin miinwaa maanaaji waabmigowin… ngii ombig owi inoodewizin agaa gichi wiisigendamowaad owi aanke bimaadiziwin wiisgendamowin- niibna maazhi doodamowin niibna aakwaadiziwin…Ngii inendizii gagwek ayaa-aan zaam ngii inendaman awii ginowaabmigo-aan bkaan aawiyaan, dibishko gonaa maandawiziyaan ado zinagiziwinan amiidash abi zhaayaan zhinda inendaagwod, nwiiji bimaadiziig ntam aawon. Giinwi kina naasaab gdo aawimi… Ndaa gagweksidoon ndo inendamowin miinwaa naagizowaabmdamaa miinwaa makwendamaa ndo maanaaji doodamowin ezhinaagwog agaa gikendamaa owi izhaa-aan ndo aanke bimaadiziwin wiisgendamowin maage ndo wiisgendamowin… “ P24*

*Wiijikiwendiwin gewe waaji piitiziimgwaa.*

Wiijikiwendiwin gewe waaji piitiziimgwa agii aawon aanji dibaachigaadeg agaa dazhinjigaadeg newen bkaan nikiiyaa. Aanin digo ayaajig dibaataanaa-aa owi niibna bemaadizijig ayaawaad miinwaa nigadendamong awii ginonigwaa owi niibna nwiiji piitiziimag aawong maanendaagwog maage onjibaamigag aanimendamowin. Aanin agii kidwog maanda goweta zengendaagwog apii ntam agii digozhinowaad, epiichi owi niish agii kidwog owi agii aawon owi memaanji gichi maanendaagwog owi nankiiwin wiinwaa gagwech.

*“Ngii aapaji aanimendam [apii agii oshki digozhinaa zhinda]. Ndo maanendaan owi wiidokaaziyaan aanimendamowin, dash maanendam owi ayaawong. Agii yaawog gichi niibna bemaadizijig.” P22*

Waaji piitiziimgwaa wiijikiwendiwin age agii dizhinjigaademigad gewe niibna aawongh mino-awii ado gikendamowin zhiwe owi nankiiwin. Agii temigad niibna dazhinjigewin owi aasgaabwitaadiwin miinwaa wiijikiwendiwin miinwaa owi izhayaawin gewe maamowi yaajig agii aawiwog inoodeziwin.

*“nakweshkwindwaa kina gewe bemaadizijig zhinda, gewe kina digo ayaajig, aapaji gwa nishin. Ngii ani aawimi owi gichi wiijiindiwaad inoodeziwin. Kina ndo kidimi gzaagin dash owi gichi mino wiijiindiwin. Waabmdowemigag owi epiichi bezhiwiziyaangh”. P17*

*“ndo nimaadibimi zhiwe gaataadibiwin miinwaa ndo ginoondimi, ndo bakwenezigemi gaataawiingh ishkode, ndo dibishko ayaawimi owi gichi inoodeziwin maampii miinwaa dowaabmdowenaa ezhiyaa-aangh miinwaa nowonj owi nikiiyaa minwaa ndo zhitoonaa owi Miskomiikan nankiiwin miinwaa gagegeti mookisemigad ndo onjibaawinaa miinwaa enji ayaaman mamaanjimigowin. Dash apii dibaatamon owi miinwaa mawiyin miinwaa moozhtowin newen inendamowinan gdo bagidendaan miinwaa woshme gdo mino zhayaa shkwaa giizhi inendamayin miinwaa kina gego. Dash owi gichi naadmaagemigad. Dibataman gego” P20*

Owi maamowi yaangh zhisijigan miinwaa gikendamowin awii apenimandaadiwin agii waabmjigaadeg aawongh naadmaadiwin e’digosingh owi nankiiwin gewe niibna.

*“…owi nendowendaagwog maamowi yaajig onjimaaigad digosingh owi ensa giizhigag enankiingh, dibishko gonaa gichitwaa ishkode, dinangidoniwin gaataabiwin apii gizhep miinwaa oshki naagshig, pana ishkode biskaneg zhiwe naami gichitwaa zhichigan dibishko aagwishinowin, um, agii gikendaan ana, kina gwa owi mashkodewaashk miinwaa sema, miinwaa wiingash miinwaa giizhkaandag meneziyaan, agii ikendaan ana, owi dibendiziwin awii zhise-aan onjike awii ayaa-aan owi bigojiwii miinwaa gikendamaa awii penimandoogwaa nwiiji piitiziimag. Uh, n’dinendam, owi nikiiyaa kina gwaya ado ayaan inaangizhewiniwaa miinwaa maagwewaad owi agaa zhitoo’aangh onjike miinwaa mika’diziyaangh. Gaawii gwaya, bakewiin newen ensa nigo namegiizhig jiibaakwe gamig nakiiwin, gaawii gwaya agii nagimigaazisii nindan nakiiwinan…aanin ndo aawimi maajitaato’aan ishkode miinwaa naaagidowendamaangh ishkode aawon owi gete bimaadiziwin nakiiwin, dibishko gonaa ishkode nyaagidowendamjig, miinwaa age aanin kwewog pane bakibidonaa’aa mashkiki miinwaa uh niin miinwaa nwiiji ninwog ndo giigoonkemi apii gashkitoyaangh’. P12*

Bezhig naasaab izhiwebizijig agii gnigzhinoog bemaadizijig baashkiji miinwaa noondaaj nisimidana epiitizijig miinwaa woshme agii yaawog minik bemaadizijig zhiwe naasaab izhiwebiziwaad dash gewe bekaanzijig. Aanin bemaadizijig zhiwe naasaab izhiwebizijig agii dazhindaanaa-aa newen e’piitizingh diwiwaag aawon maanenjigewin miinwaa dibaataan-aa owi bakaaniyaawaad zhiwe woshme gechi piitizijig maamowi yaawaad miinwaa woshme eshkiniigijig maamowi yaawaad. Aanin gewe bemaadizijig nondaaj nisimidana epiitizijig agii izhayaawog gewe woshme e-piitizijig gaawii agii minaadenimigsii-aan zaam onji epiitiziwaad miinwaa age izhayaawog aangwadinong agii wiindamaaagwaan awaa zhichigewaad. Aanin gewe bemaadizijig woshme nisimdana epiitizijig agii dibaataanaa-aa gaawii mashi nitaawigisiwaad aanin woshme eshkiniigijig bemaadizijig aawon maanenjigewin nendowendaagwog zhiibendamowin. Owi nankiiwin agii aandendamawog awii bakeshimaa-aad gewe ezhi piitizingh maamowi yaajig niigaan aawong.

Miinwaa aanke ninda dibaajimowinan e’dazhinjigaadeg, owi aanji gagwejimindwaa aanin digo ayaajig agii kidwog agii dowendamowaad woshme awii digokaaziwaad owi Giizhaa Agaa Yaajig endinakiiwin dash gaawii agii gashkitosiinaa-aa mashi zaam newen COVID-19 nigaasijigewinan. Niibna agii wiindamaagewog owi agii nakiiwog nikiiyaa maage agii aanjitonaa-aan ado nda gashkitowinan dibishko gonaa aabajitaawaad maage dowaabmdamiwaad oshki nakiitaagewin maage zhaawaad ishpi gikinomaage gamig:

*“Ngii aandaakonige ngoji owi ishpi gikinomaagewin…ngii dapinigwo zhiwe [ishpi gikinomaage gamig] owi Giizhaa Agaa Yaajig mina yaawin miinwaa maanjiminigowin gikinomaadiwin. Dash gnimaa ndaa nankii zhiwenjige naakiiwin maage wiiji naakiimiwaa eshkiniigijig awii nigaashimagwa awii azhaawaad owi bimasewin agaa zhaa-aan. Ndo inendam owi ishpi gikinomaagewin aawon memaanji nishing anikiiyaa azhaangh.”* P19

*Nigodwaaswi giizis aanji gagwejimindwaa*

Ngii zhitonaanin nigodwaaswi giizis aanji gagwedwewinan owi 20 gewe 39 (51.2%) nda gikenjigewin digo ayaajig. Gaawii gewe 20 digo ayaajig agii wiindamaagewog gaawii agii nikaazisiinaa-aa aaboon owi apii nigodwaaswi giizis aanji gagwedwewinan. Naanon gewe 20 (25%) agii kidwog agii bagishinowaad ngoji apii agii azhesewin owi apii shkwaa maajaawaad owi nankiiwin dash gimaawipii agii boonitaawog.

Gegaa kina digo ayaajig agii kidwog agii mina maajiishkaawog ado inendamowin nigodwaaswi giizis shkwaa owi akiingh temigag noojimowin nankiiwin agii ekwaasemigag, aanawi gwa agii tenoon ombendamong miinwaa dibasendamong. Aanii agii wiindamaagewog maanendamowinan owi aanji digoyaangh, dibishko gonaa apii ne-aab agii giiwewaad odi e-gaaching odena zhiwe aazhigwa wiijikendiwinan agii digosin nibewaabo nikaazang. Aabaji temigad aasgaabiwitaagewin onji Gagwekwaadziwin Miikan agii dazhinjigaademigad zhiwe aanji gagwedwewin miinwaa agii gichi inendaanaa-aa gewe digo ayaajig, dash bezhig agii mkaan maanda agii maanendaagwog apii shkwaa maajaad owi nankiiwin.

*“Ngii mooshitoon gichi mashkowi inendamowin owi webinigowin. Gdo zhitoon owi wiijiindiwin owi akiingh enji temigag gagidomaagejig miinwaa geskana kina owi agii maajaamigad. Nigaasemigad. [Odena giizhiitaamigag naagidowenjigewin gagidomaaged] agii zhiibinketaage miinwaa owi nishin dash gaawii naasaab aawizino newen wiijikiwendiwinan agaa zhitowin newen niswi giizis zhiwe akiingh. Bkaan makigaadegin.* P18

*Bkaan makigaadegin.*

Digo ayaajig age agii kidwog owi akiingh enji temigag nankiiwin miigsemigad gewe bemaadizijig e’zhiitaawaad awii wiidokaaziwaad, owi, gaawii ayaasiwaad owi nibewaabon nikaaziwaad. Aanin digo ayaajig nandawendamoog woshme minik ayoongh owi Misko Miikan miinwaa agii makaanaa-aa owi aawongh memaanji naadmaagemigag owi nankiiwin. Aanin digo ayaajig agii nandawendaanaa-aa woshme zhisijigaadeg bebezhig ginonaa-aad gagidomaagejig dash owi aabdeg gagwedwewaad bebezhig ginonaa-aad minik wiinwaa.

*Enkiitaagejig gagwejimigaaziwaad*

Nishwaaswi enkiitaagejig agii gagwejimigaaziwog owi gikendamowiniwaa aawiwaad Gwekwaadiziwin Miikan enkiitaagejig miinwaa ado gikendamowin agii maandobiigaadenon ekwaawiingh maanda bakebiigan. Apii dazhinjigaadeg onji owi wiidokaaziwaad owi Gwekwaadziwin Miikan, enkiitaagejig agii dibaataanaa-aa owi ombigiwaad owi Giizhaa Agaa Yaajig inodewiziwin miinwaa dowendamowaad awii maadokiiwaad inaadiziwin maage gikendamowin gewe nankiiwin digo ayaajig miinwaa biskaabi miigwewaad owi odena; ombigwaad owi bakeshinowaad ado Giizhaa Agaa Yaajig inaadiziwin miinwaa owi aki miinwaa dowendamowaad awii aanji dikobizowaad miinwaa woshme gikendamowaad owi Giizhaa Agaa Yaajig onjibaawaad; aazhigwa gikendamowin newen agojiingh miinwaa aki enji temigag dinakimigiziwinan (dibishko bimakwazhiwe jiimaan gikinomaaged, gabeshii wiigwaam gagidomaaged) miinwaa dowendamowaad awii nakiiwaad akiingh; zhaazhigwa zhiwebiziwin owi inendamowin mino yaawin miinwaa mamaanjigowin; owi izhi’gagaandind owi nankiiwin ado wiijikiwewinan maage inodewiziwin; miinwaa ombigid zhiwe gaachi odena odi Manidoo Minis miinwaa izhi’gagaandinind zhiwe gewe megwaa iyaajig enkiitaagejig. Gewe enkiitaagejig agii miigwenaa-aan aanin aanjichigewinan dibishko gonaa giikinigaadeg nendowendaagwog gikinomaadiwin owi inendamowin mina yaawin miinwaa mamaanjimigowin gaginoondiwin miinwaa woshme gikinomaadiwin owi gabeshii wiigwaam iniwendaagwog miinwaa agojiingh nakiiwin nigajiiwin (dibishko gonaa, nikaazong owi jiibaakwe gizhaabkizigan). Miziwe gwa, enkiitaagejig agii wiindamaagewog gikendamowinan wiiji nakiingh owi nankiiwin.

**AANIKANOOTAJIGAADEG**

Owi gnigsijigaademigag nikiiyaa zhichigewin nda gikenjigewin agii makaanaa-aa owi akiingh enji temigag noojimowin nankiiwin onji Giizhaa Agaa Yaajig bemaadizijig eyaamiwaad inendamowin mino yaawin miinwaa waaboon nikaazangh maanendaagwog agii gagaandinaan dikobizong owi Giizhaa Agaa Yaajig Bimaadiziwin miinwaa owi aki. Dikobizong owi aki agii waawiinjigaademigad owi bezhig memdage nenda gashkitoongh gewe digo ayaajig agii bigosendamog awii gashkitowaad onji owi akiingh enji temigag noojimowin nankiiwin, miinwaa newen aanke gagwedwewinan, niibna agii kidwog maande agii gashkichigaademigad. Newen dibaabajigewin miinwaa dibaginjigewin agaa makigaademigag agii nikaazam niibidesijigaadeg awii miigwengh mooshkin nisastamiwin owe nankiiwin gashkitowin. Apii dibaabajigewinan agii dibaginjigewin debijigaademigad digo ayaajig gikendamowiniwaa miinwaa inenjigaadeg gashkitowinan owi nankiiwin, dibaginjigewinan ezhiwebak ninda makigewinan owi maandobiigaademigag nankiiwin giizhitoongh miinwaa nibe aaboon nikaazangh apii aanji gagwedwewin. Ninda makigaadewinan gagwekaabiigisingh, owi aabitaa digo ayaajig agaa giizhitowaad aanke gagwedwewin nigodwaaswi giizis shkwaa giizhiitaangh owi nankiiwin agii wiindamaagewog gaawii ayaa-aabi ado nikaazisiinaa nibe aaboon.

Ndo makigaadewinan gagweksinoon owi aazhigwa nda-gikenjigewin owi inenjigaademigag owi dikobizowin owi aki aawon nendowendaagwog owi Giizhaa Agaa Yaajig mino yaawin, miinwaa newen inendamowin mino yaawin nanaawisijigewinan nanaagwisingh owi dikobiziwin owi aki miigsenoon awii nikaazang zhiwe Giizhaa Agaa Yaajig odenwinan (Ahmed et al., 2023b; Walsh et al., 2020). Aazhigwa nda-gikendamowinan agii waabmdowemigadoon newen akiingh enji temigag bimaadizingh miinwaa manidokewin zhichigewinan mino doodaagemigad odeniwingh wiidokaaziwin miinwaa age jichaag, wiiyaw, izhayaawin, miinwaa inendamowin mino yaawin gewe Giizhaa Agaa Yaajig netaawigijig (Ahmed et al., 2021; Walsh et al., 2020). Apii maanda nda-gikenjigewin, apii owi aanke gagwejimigewin, aanin digo ayaajig agii gagwek kidwog owi dowendaanaa-aa awii woshme wiidokaaziwaad ado odenwingh miinwaa gegaa gwa kina agii dazhindaanaa-aa naawisijigewinan owi jichaag, izhayaawin, miinwaa inendamowin mino yaawin owi nikiiyaa dikobizaowaad owi Giizhaa Agaa Yaajig bimaadiziwin miinwaa owi aki. Gwekwaadziwin Miikan bamibidemigad zhiwe bigodkamig, dash akiingh enji temigag noonjimowin agii waabmdowemigad awii bamitaagemigag gewe Giizhaa Agaa Yaajig eshkiniigijig zhiwe gichi odenwingh age. (Hatala et al., 2020). Noojimowin owi inaadiziwin aawongh e’nishingh gikinowaabnjigan onji mamaanjimingowin noojimowin (Dobson & Brazzoni, 2018).

Owi gaawii zhisijigaadesinag ginowaabmdamiwin nda gikenjigewin gaawii adaa waabmdowesiin enji zhiwebak miinwaa owi aanji gagwedwewin minik dibasaamigad. Gagwejimigewinan agii tenoon owi ayaawaad Gikendamowin Genwendamajig agaa aasgaabiwitomowaad owi nankiiwin miinwaa maanda gnimaa adaa madookinon digo ayaajig nakwetamowiniwaan. Gewe nenda gikenjigejig dibaabiishkodamowaad owi dibagenjigewin gaawii agii aawisiiwog Giizhaa Agaa Yaajig maage gechi gikendaasojig owi Giizhaa Agaa Yaajig mina bimaadiziwin owi gnimaa agaa madookidaagemigag owi dibagenjigewin maandonigaadeg miinwaa dibaabiishkojigaadeg. Makigewinan agii dazhinjigaadenon gewe Gechi Piitizijig miinwaa Gikendamowin Genwendamjig awii debwechigaadeg miinwaa owi ezhiwebak ndo aankanotamawinan. Owi gnigsijigaadeg nikiiyaa nda-gikenjigewin newen gikendamowinwaa gewe digo ayaajig zhiwe akiingh enji temigag noojimowin nankiiwin onji inendamowin mina yaawin miinwaa mamaanjimingowinan miigwemigad gagwek waabmdamiwinan onjibaamigag owi gichi niibna dibagenjigewin miinwaa miigwemigad waabmdamiwinan newen gnimaa age nikaazong awii wiindamaagengh akiingh enji temigag noojimowin nankiiwinan.

*Giizhendamowin*

Owi akiingh enji temigag noojimowin nankiiwin onji Giizhaa Agaa Yaajig eshkiniigijig miinwaa netaawigijig eyaamwaad inendamowin mina yawing miinwaa mamaanjimingowin maanendaagokin miigwemigad dikobizowin owi Giizhaa Agaa Yaajig bimaadiziwin miinwaa owi aki, miinwaa gewe digo ayaajig agaa giizhitowaad owi aanke gagwedwewin gaawii agii nikaazisiinaa-aan nibe mashkikiin. Akiingh enji temigag noojimowin nankiiwinan adaa maajitaachigaadenon awii naazhinigaadeg newe wiisigitowinan owi oshki digoshinajig gagaandinigaadeg ado bimaadiziwiniwaa owi digosingh owi maage aanke newen aasgaabiwichigaadegin e’digosin nibe-aaboon owi meshkod naandawiwewin. Giizhaa Agaa Yaajig bemaadizijig adaa bagidinimaagaaziwog akiing enji temigag noojimowin nankiiwinan miinwaa mina apii misendamowin owi noojimowin adaa gagaandinigaademigad owi aasgaabiwichigaadeg megwaa etmigag miinwaa oshki nankiiwin owi aanke ne-aab miigwengh aki gewe Giizhaa Agaa Yaajig bemaadizijig aawongh bezhig owi ishko oshki digoshinajig gagaandinigewinan.

**Miigwewin owi Gikendaasowin**

● Wegonesh maanda nda-gikenjigewin e’aankesidod owi megwaa etemigag Gikendaasowin?

Maanda nda-gikenjigewin miigwemigad moshkin ginowaabmdamiwin newen gikendamowinan gewe digo ayaajig zhiwe akiingh enji temigag noojimowin nankiiwin onji Giizhaa Agaa Yaajig bemaadizijig eyaamiwaad inendamowin mina yaawin miinwaa nibe mashkiki nikaazongh maanenjigewinan. Ndo makigewinaa ishpisidoon owi gichi piitendaagwog owi dikobiziwin owi Giizhaa Agaa Yaajig bimaadiziwin miinwaa owi aki owi gagaandinigaadeg mino ayaawin miinwaa mino bimaadiziwin. Miinwaa aanke, owi nda-gikenjigewin gikinwaajitoon owi gichi inendaagwog owi miigwench inaadiziwin nowendaagwog bakaanag owi gagwech dnowaa noojimowin zhichigewinan, aasgaabiwichigaadeg owi woshme maajiishkaachigaadeg newen akiingh enjig temigag miinwaa inaadiziwin naanaagwisingh noojimowin nankiiwinan.

● Wegonesh newen ishpi dodaaagemigag owi bigo iwiyaajig mino yaawin nigaasijigewinan, izhichigewin maage naaknigewin?

Giizhaa Agaa Yaajig bemaadizijig adaa yaanaa-aa misendamowin newen akiing enji temigag noojimowin naankiiwin. Maanda adaa gagaandinaan mino yaawin gagwechgwa miinwaa memdage gewe bemaadizijig eyaamjig inendamowin mino yaawin miinwaa nibe aaboon nikaaziwin maanendamowinan.

**Gikenjigewin Onjibaamigag**

Ahmed, F., Liberda, E. N., Solomon, A., Davey, R., Sutherland, B., & Tsuji, L. J. S. (2023a). Indigenous Land-Based Approaches to Well-Being: Owi Niska (Goose) Zigakinigengh Nankiiwin odi Subarctic Ontario, Ganaada. *International Journal of Environmental Research and Public Health*, *20*(4), 3686. https://doi.org/10.3390/ijerph20043686

Ahmed, F., Liberda, E. N., Solomon, A., Davey, R., Sutherland, B., & Tsuji, L. J. S. (2023b). Giizhaa Agaa Yaajig akiingh enji temigag zhichigewinan owi mino bimaadiziwin: Owi Sibi (River) nankiiwin odi Subarctic Ontario, Ganaada. *International Journal of Circumpolar Health*, *82*(1), 2252595. https://doi.org/10.1080/22423982.2023.2252595

Ahmed, F., Zuk, A. M., & Tsuji, L. J. S. (2021). The Impact of Land-Based Physical Activity Interventions on Self-Reported Health and Well-Being of Indigenous Adults: Owi Zhichigewinan Nda Gikenjigaadeg. *International Journal of Environmental Research and Public Health*, *18*(13), 7099. https://doi.org/10.3390/ijerph18137099

Cunsolo Willox, A., Harper, S. L., Ford, J. D., Landman, K., Houle, K., Edge, V. L., & Rigolet Inuit Community Government. (2012). “From this place and of this place:” climate change, sense of place, and health in Nunatsiavut, Canada. *Social Science & Medicine (1982)*, *75*(3), 538–547. https://doi.org/10.1016/j.socscimed.2012.03.043

Dobson, C., & Brazzoni, R. (2018, July 16). *Akiingh enji temigag noojimowin: Carrier First Nations’ Addiction Recovery Program – Te Mauri – Pimatisiwin*. https://journalindigenouswellbeing.co.nz/journal_articles/land-based-healing-carrier-first-nations-addiction-recovery-program/

Gittings, L., Malama, K., Logie, C., Lys, C., Taylor, S. B., McNamee, C., Mackay, K. I., & Admassu, Z. (2024). Peer and land-based approaches for fostering empowering and healthy relationships with Indigenous and northern young people in the Northwest Territories. *PloS One*, *19*(4), e0298166. https://doi.org/10.1371/journal.pone.0298166

Groenewald, T. (2004). Owi Debwewong Nda Gikenjigaadeg Mazininigan Waabmdowengh. *International Journal of Qualitative Methods*, *3*(1), 42–55. https://doi.org/10.1177/160940690400300104

Hatala, A. R., Njeze, C., Morton, D., Pearl, T., & Bird-Naytowhow, K. (2020). Akiin miinwaa agojiingh aawong enjibaamigag owi mino yaawin miinwaa basabaagiziwin gewe Giizhaa Agaa Yaajig eshkiniigijig zhiwe gichi odena Canadian ezhiwebak. Owi mazinaaziwin-giigidowin ginowaabmjigaadeg. *BMC Public Health*, *20*(1), 538. https://doi.org/10.1186/s12889-020-08647-z

*Home*. (n.d.). Gwekwaadziwin Miikan. Retrieved March 31, 2023, from https://gwek.ca/

Jonas, D. E., Amick, H. R., Feltner, C., Bobashev, G., Thomas, K., Wines, R., Kim, M. M., Shanahan, E., Gass, C. E., Rowe, C. J., & Garbutt, J. C. (2014). Mshkiki nikaazong gewe nitaawigijig ayaamiwaad ishkode-aabo aakoziwin zhiwe zaagiji aakoziigamig ayaawaad: Owi Zhichigewin nda-gikenjigaadeg miinwaa maamowi dibaabijigaadeg. *JAMA*, *311*(18), 1889–1900. https://doi.org/10.1001/jama.2014.3628

Klimas, J., Fairgrieve, C., Tobin, H., Field, C.-A., O’Gorman, C. S., Glynn, L. G., Keenan, E., Saunders, J., Bury, G., Dunne, C., & Cullen, W. (2018). Inendamowin-wiijiiwin nanaaginiwewinan awii naazhinigaadeg ishkode-aabo miinikwengh zhiwe neniish zinagiziwin ishkode-aabo miinwaa begidinigaasinag nibe mashkiki enkaazijig. *The Cochrane Database of Systematic Reviews*, *12*(12), CD009269. https://doi.org/10.1002/14651858.CD009269.pub4

Mattick, R. P., Breen, C., Kimber, J., & Davoli, M. (2014). Buprenorphine nikaazongh meshkod wezhige mashkiki maage methadone nikaasongh onji nibewaabo dowendamong. *The Cochrane Database of Systematic Reviews*, *2*, CD002207. https://doi.org/10.1002/14651858.CD002207.pub4

Morton Ninomiya, M. E., Tanner, B., Peach, L. J., George, N., Plain, S., George, T., Graham, K., Bernards, S., Brubacher, L. J., & Wells, S. (2022). Anishinaabeg onjibaamigag mashkoziiwin: Gikendamowin onji Inakaaneziwinan bemaadizijig agaa gikendamowaad indendamowin mino yaawin miinwaa nibewaabi nikaaziwin maanendaagwog. *Social Science & Medicine (1982)*, *309*, 115197. https://doi.org/10.1016/j.socscimed.2022.115197

National Inquiry into Missing and Murdered Indigenous Women and Girls (Canada), Buller, M., Audette, M., Eyolfson, B., & Robinson, Q. (2019). *Aanji debinamong mashkowiziiwin miinwaa endinakiingh: The final report of the National Inquiry into Missing and Murdered Indigenous Women and Girls*. https://www.mmiwg-ffada.ca/final-report/

Nejade, R. M., Grace, D., & Bowman, L. R. (n.d.). Wegonezh owi endodaagemigag agojiingh owi mina bimaadiziwin? Owi zhiibaamijigaadeg nda gikenjigewin newen zhibiiganan. *Journal of Global Health*, *12*, 04099. https://doi.org/10.7189/jogh.12.04099

Nelson, S. E., & Wilson, K. (2017). Owi inendamowin mino yaawin gewe Giizhaa Agaa Yaajig bemaadizijig maampii Ganaada: Owi dibaamjigaadeg owi nda-gikenjigewin. *Social Science & Medicine (1982)*, *176*, 93–112. https://doi.org/10.1016/j.socscimed.2017.01.021

Redvers, J. M. (2016). *Land-based Practice for Indigenous Health and Wellness in Yukon, Nunavut, and the Northwest Territories*. http://hdl.handle.net/11023/2996

Shaughnessy, P. (n.d.). *Whitepath Consulting home of TheRedPath Programs*. Whitepath Consulting Inc. Retrieved March 31, 2023, from https://whitepath-consulting-inc.myshopify.com/

Shaughnessy, P. (2001). *FRICTION WITHIN THE MACHINE: ABORIGINAL PRISONERS BEHIND THE WALL*. Trent University. https://www.nlc-bnc.ca/obj/s4/f2/dsk3/ftp04/MQ57996.pdf?is_thesis=1&oclc_number=81213122

Van Zee, A. (2009). The promotion and marketing of oxycontin: Commercial triumph, public health tragedy. *American Journal of Public Health*, *99*(2), 221–227. https://doi.org/10.2105/AJPH.2007.131714

Walsh, R., Danto, D., & Sommerfeld, J. (2020). Akiingh Enji Temigag Nigaasijigewin: A Qualitative Study of the Knowledge and Practices Associated with One Approach to Mental Health in a Cree Community. *International Journal of Mental Health and Addiction*, *18*(1), 207–221. https://doi.org/10.1007/s11469-018-9996-3

**Niibidebiigan 1.** Digo Ayaajig iyaawiwaad (N=39)

| **Iyaawiwaad:** | **n (%)** |
| --- | --- |
| Kwewog, Ninwog | 18 (46), 21 (54) |
| Epiitiziwaad (biboonan) |  |
| 18-30 | 24 (61) |
| 31-40 | 8 (20) |
| 40+ | 7 (18) |

Makakoons 1. Dikobizowin owi Giizhaa Agaa Yaajig Bimaadiziwin

Aanji dikobizowin:

*“Ngii gikendaan nikiiyaa awii waawiindiziyaan, e’gaaching kidiwinan maampii miinwaa odi owi e’nishingh. Miinwaa goweta owi inaadiziwin zhiwe kina enakiiyaangh, dash digosijigaademigad. Ndo gageti anii biskaabii ne’aab owi jichaag owi agaa ayaamaa mewizha dibishko apii Nokomis agii bamiyaad.” P2*

*Ngii ombig zhiwe shkoniganingh miinwaa ngii azhaa owi gichi inaadiziwin gikinomaage gamig…Dash ngii gikendaan bangii owi inaadiziwin, dash maanda ngii gagaandinigwon gikendamaa owi gaawii aapaji zhichigesiim. Aapaji gwa zhe’aakosin gaawii nipaajiwii nikiiyaa zhichigewin- tenoon nowoonj dnowaa nikiiyaa eshichigengh miinwaa ndo maajitaa gikendamaa debinaag gwa debwewendaman owi Gizhe-manidoo maage gdo ishpenimad owi gaawii gego gdo zhichigesii myaanaadag. Miinwaa ngii miigwon woshme niibna gikendamowin owi ndo inaadiziwin. Zaam owi gichi niibna biboon ngii aanjii ngoji bkaan endaayaan miinwaa inendaan owi agaa onjibaayaan.” P16*

Nakiitamaa owi digobiza’aan ndo inaadiziwin agii naadmaaginaa-aa niibna digo ayaajig owi ado mino inendamowin miinwaa dikobinigan bimasewinan. Gashkitoongh awii wiidokaazangh zhiwe inaadiziwin zhichigewinan agii kidwog agii naadmaagemigag.

*“Agii gikendaan ana, ngi ayaan besha ayaa-aan aanin gechi piitizijig owi ndo bimaadiziwin…dash ngii makaan owi ayaa-aan zhinda owi zhichigryaan kina gego nigodingh agaa gikinomaagoyaan awii zhichigeyaan dash gaawii ngii zhichigesii zaan ngii awanishin zhiwe ndo mamaanjimingowinan, maampii ndo moozhitoon gegwa eniwek aakwendamowin miinwaa ndo dowendiziin awii mooshitoo-aan miinwaa nimakwendaan gego gechi maanaadag miinwaa ndo dowendaan awii gajibowe-aan newen inendamowinan, ngii gikendaan owi gajitoyaan aawii meshkodsido-aan nibe aaboon miinwaa ishkode-aabo newen ninda mashkikiin eyaamaangh zhinda awii nikaaziyaangh zhinda. Nga izhaa miinwaa bagidinaan aanin sema zhiwe ishkode miinwaa bakwenezige miinwaa nga bakibinaa aanin giishkaandig miinwaa nga zhitoon giishkaandig nibiish miinwaa nga giziibiigizhe miinwaa nga minikwen aanin giishkaandig aabo miinwaa nga bibaamse ngoji bkaan owi miikaans miinwaa gnimaa nga awanishin miinwaa nimadab zhiwe aki jiigi mitig maage asin miinwaa aanji dikobinidis. Miinwaa ngii gashkitoon minadenjigewin miinwaa gichi inendamaa owi nikiiyaa ninda kina owi gechi piitizijig agii wiindamaagiyaan awii dowendaagwog awii maajitaayaan gegeti adaa nakiimigad miinwaa ndo dowendaan owi agii daapinamaa woshme getinaamendamaa niibna biboongag zaam ndaa megwaach ayaa woshme e’nishingh nongo megwaa.” P12*

Gikendamowin owi inaadiziwin:

Dowendamaa awii gikendamaa woshme miinwaa awii dikobiso-aan agii gichi piitendaagwod owi gikendiziiyaan.

*“…Gaawii gego ngii gikendiziin owi ndo bimaadiziwin agaa yaawong, gaawii ngii gikendiziin, aanin bemaadizijig maampii giikendaanaa ado dodem, giikendaanaa-aa ezhi giigidowaad ado iniwewin, miinwaa gaawii gego ngii gikendiziin, naa.* *Nigaa’endaagwod.* *Owi nendaagwod gaawii gego ndo ayaanziinon onjibaawin dinendam, maage gaawii giikendiziin ayaayin, naa.* *Dash ngii gikendaan woshme…”P5*

Aanin digo ayaajig agii dazhindaanaa-aa bigosendamowaad awii aanji dikobizowaad owi wiinyaa ayaawiwaad owi digo ayaawaad zhiwe akiingh enji temigag noojimowin nankiiwin.

*“Ngii dowendaan ne-aab awii gikendiziiyaan miinwaa mikodiziiyaa miinwaa miidash agii nendamaa maanda adaa aawon memaanjig nishingh zhiwe awii zhichige-yaan owi…Ngii bigosendam awii gikendamaa woshme gego owi ndo inaadiziwin miinwaa niin ayaawiyaan” P6*

*“…Ndo gajitoon awii makaamaa owi ayaawiyaan…aanawi ngii gikendaan ayaawiyaan, dash ndo gajitoon ne-aab awii aawiyaan nendowendamaa” P15*

*“Apane gwa agii maajitaayaan minikweyaan, ngii aanjigwon. Ngii naajitoon ayaawiyaan miinwaa niin. Ngii naajitoon ndo inendamowin miinwaa ngii naachidis” P1*

Makakoons 2. Dikobizowin owi aki

*“Nongo apii owi bagwadkamig [agii gichi naadmaagemigad etemigag owi nankiiwin]. Ayaangh odi aki aapaji gwa naadmaagemigad…ngoji bkaan ayaayin gewe ‘gwiijikiwenig’ .”P8*

*“Ndo izhayaa niin onji, ombigiyaan Ngii gwiiniwendam owi inaadiziwin ayaawiyaan. Um, miinwaa niibna newen zinagiziwinan agaa nakweshkamaa zhiwe ndo bimaadiziwin agii aawon tesinag zhinomaagewin, dash ndo debwewendam abi zhaayaan zhiwe aki nimaajiinigwon ne-aab owi dikobizowin owi Ashkaakamig Kwe miinwaa miinigwon owi zhisewin awii gikendamaa woshme newen inaadiziwin naagijigewinan miinwaa gikinomaagewinan…dikobizong owi aki ndo inendam gmaajiinigwon ne-aab niibna newen gichitwaawendaagokin. Gagwejiiwin gwiiyaw wiidokaaziimigag zhiwe inendamowin, gdo inendamowin mashkoziiwin, izhayaayin miinwaa jichaag…goweta biskaabiiyin odi agaa onjibaayin. N’dinendam tenoon niibna gego gaawii gdo nisidiwendiziinaanin. Dash n’dinendam manjigwa maage gaawii nakweshkiziiyaan, temigad niibna nakiiwin owi jichaag goweta owi dikobizowin ne-aab Ashkaakamig Kwe.” P12*

*“…zhiwe wegodogwen bkaan noojimowin wiigwaam maage kowe dinizingh wiigwaam maage noojimowin wiigwaam agii ayaa-aan Ngii ayaa gagwech besha onji owi ishkodewaboo endinadaandingh maage n’sonigowin wiigwaam, miidash maampii aabdeg anii miigwen nishwaaswi giizhigad wiindamaagewin awii ani maajaayaan miinwaa ngii gikendaan giishpin owi nankiiwin zinagag deminig owi izhayaa-aan awii gijiboweyaan miinwaa azheseyaan, adaa temigad owi aandendamowin biinji newen nishwaaswi giizhigad biitwi owi apii agaa miigweyaan wiindamaagewin miinwaa owi apii gegeti agaa ani maajaayaan owi megwaach adaa aandendam.” P12*

Waaji ayaajig age dibaataanaa-aa ishpaamigag owi akiing enji temigag noojimowin aawong meshkod newen bkaan nojimowin temigag dibishko gonaa gikinomaage gamig maage aakoziigamig.

*“Owi onji ngii dowendaan awii zhitoyaan owi akiingh enji temigag agii aawon gaawii dowendiziiyaan awii zhichigeyaan gego owi mashkikii nikaaziwin…agii gikendaan ana nimadibiiyaan zhiwe nibomigag aajikinigan temigag nibomigag waaskonenjigan miinwaa gdo debinaanon niibna gagwedwewinan mokiitaagiyin miinwaa aanin gego.” P9*

*“…ngii abi niindaagwo waasamo biiwaabik mazina’igan temigag nowonj wenaamjigewinan owi noojimowin, dash ngii ginowaabmdaanon waasamo asabi mazina-iganon miidash maanda goweta agii aawon akiingh enji temigag. Ngii aabaji mina zhayaa ayaa-aan agojiingh. Ngii gwiiniwi adaa minik niswi biboon, miinwaa uh, ngii dinis megwe matigwaaki…ngii onendam abi zhaayaan maampii nda ayaa agojiingh, gaawii aawizio gikinomaage gamig onjibaamigag agaa indendaama adaa zhigadendaagwod miinwaa owi, zinagad awii nimadibiiyaan bekaa zaami gnesh…Miinwaa agaa kidiyaan owi inaadiziwn ayaawong michaamigag niin onji. Ngii inendam owi adaa temigad woshme noojimowin awii gashkitoyaan owi nikiiyaa ayaawiyaan woshme nda mayaawisidoon dash owi nibadibiiyaan gikinomaage gamig.” P5*

Makakoons 3. Wiijikiwendiwin gewe waaji piitiziimgwaa.

*“kina ndo nibadimimi gaataawiingh miinwaa dibaataanaanim izhayaa-aangh miinwaa ndo aasgaabiwitaadimi dash owi aawon memaanji ishpaamigag owi age. Ndo dibaataanaa nowonj gego maamowi miinwaa nigodingh bezhi gwaya maadookiid miidash kina ndo maadookiimi miinwaa aapaji gwa gichi wiingaamigad. Miinwaa gdo bagidinaanon newen kina izhayaawinan miinwaa owi kina wiisigendamowin miinwaa nowonj gego agaa dodaagiyaan mamaanjimingowin newen nibe-aaboon maage wegwen gwa owi ishkodewaabo miinwaa nowonj gego. Dash apii dibaatamon owi gdo woshme mino azhayaa. Gnimaa gwa gdaa maanaaji zhayaa ajina dash shkwaa giizhi dibaatamon gdo maamiikwa izhayaa. Dash owi aawon mino-yaawin.” P12*
